# Further evidence for functional recovery of AQP2 mutations associated with nephrogenic diabetes insipidus

**DOI:** 10.14814/phy2.14866

**Published:** 2021-06-13

**Authors:** Pierre Bissonnette, Yoann Lussier, Jessica Matar, Alexandre Leduc‐Nadeau, Sandra Da Cal, Marie‐Françoise Arthus, Robert J. Unwin, Julia Steinke, Dharshan Rangaswamy, Daniel G. Bichet

**Affiliations:** ^1^ Département de Pharmacologie et Physiologie Université de Montréal Montréal QC Canada; ^2^ Centre de Recherche Hôpital du Sacré‐Cœur de Montréal Montréal QC Canada; ^3^ Department of Renal Medicine University College London London UK; ^4^ Division of Pediatric Nephrology Helen DeVos Children’s Hospital and Clinics Grand Rapids MI USA; ^5^ Department of Nephrology Kasturba Medical College Kasturba Hospital Manipal Academy of Higher Education Manipal Karnataka India

**Keywords:** aquaporin‐2, functional recovery, nephrogenic diabetes insipidus, recessive mutations

## Abstract

Aquaporin‐2 (AQP2) is a homotetrameric water channel responsible for the final water reuptake in the kidney. Disease‐causing *AQP2* mutations induce nephrogenic diabetes insipidus (NDI), a condition that challenges the bodily water balance by producing large urinary volumes. In this study, we characterize three new *AQP2* mutations identified in our lab from NDI patients (A120D, A130V, T179N) along the previously reported A47V variant. Using *Xenopus* oocytes, we compared the key functional and biochemical features of these mutations against classical recessive (R187C) and dominant (R254Q) forms, and once again found clear functional recovery features (increased protein stability and function) for all mutations under study. This behaviour, attributed to heteromerization to *wt*‐AQP2, challenge the classical model to NDI which often depicts recessive mutations as ill‐structured proteins unable to oligomerize. Consequently, we propose a revised model to the cell pathophysiology of AQP2‐related NDI which accounts for the functional recovery of recessive AQP2 mutations.

## Graphical abstract

Mutations to AQP2 induce nephrogenic diabetes insipidus (NDI), where low/inactive water channels cause large urinary waste of possible fatal outcome. Our past studies have shown that several AQP2 mutations could be functionally recovered by associating to wild‐type AQP2 (*wt*‐AQP2) into the protein complex (tetramer). This study presents the characterization of three new AQP2 mutations from actual case‐studies in regard to recovery capacities, and conclude by proposing a revised pathophysiological model to NDI, one where AQP2 mutations are now presented as mostly recoverable through *wt*‐AQP2 association into functional water channels.

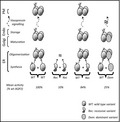

## 1 INTRODUCTION

Aquaporin‐2 (AQP2) is a water channel specifically expressed in principal cells of the kidney collecting duct. Structurally similar to all known aquaporins, AQP2 is constituted of four identical subunits, each one bearing its own functional pore. Its activity, central to the regulation of body water homeostasis, is under the tight control of the antidiuretic hormone vasopressin, which regulates the apical expression of the channel (Christensen et al., 2000; Katsura et al., 1995; Knepper & Inoue, 1997; Nielsen & Agre, 1995). Natural mutations in AQP2 are responsible for nephrogenic diabetes insipidus (NDI), a condition where affected individuals are unable to concentrate ultrafiltrate, thus producing large urinary volumes, with corollary health problems including polydipsia, polyuria, dehydration and possibly death (Bichet, 2020; Bockenhauer & Bichet, 2015).

Previous studies, using mostly heterologous expressing systems such as *Xenopus laevis* oocytes and cell lines, have described the key features of NDI‐causing AQP2 mutations in terms of reduced synthesis efficiency (Guyon et al., 2009; Marr, Bichet, Hoefs, et al., 2002), inadequate synthesis (quality control/maturation) and routing (Deen et al., 1995; Lin et al., 2002; Marr, Bichet, Hoefs, et al., 2002). Most AQP2 variants described so far were labelled as recessive (*rec*) since only *rec/rec* homozygotes were affected leaving *wt/rec* heterozygotes to be asymptomatic (Robben et al., 2006). Globally, the mutated protein is said to be misfolded, prompting intracellular retention mostly in endoplasmic reticulum with ensuing premature degradation (class II mutations; Robben et al., 2006). These mutations are found to be disseminated throughout the protein's structure which constitutes the channel per se, with exception of P262L (de Mattia et al., 2004). On the other hand, the few dominant (*dom*) mutations identified so far were shown to be restricted to the intracellular C‐terminal end of the protein (S216F is most N‐ter variant; Moon et al., 2009), in a segment not participating to the channel structure but instead related to regulatory features (plasma membrane targeting; Schenk et al., 2005). Consequently, and conversely to *rec* forms, *dom* mutations retain their ability to associate with *wt*‐AQP2, inducing NDI phenotypes even in heterozygotes through sequestration of *wt/dom* heteromeric complexes and *dominant negative effect* (DNE) actions (Tamarappoo & Verkman, 1998). These key features have led to a general model for AQP2‐dependent NDI where subunit interactions within the tetrameric structure of the channel are central (Kamsteeg et al., 1999; Robben et al., 2006).

However, our recent work describing *rec*‐AQP2 mutations of mild phenotypes not only stressed that such mutations were not actually ill‐structured but most importantly demonstrated their capacity to associate with *wt*‐AQP2 within structured channels, recuperating function and proper targeting in the process (El Tarazi et al., 2016; Guyon et al., 2009; Leduc‐Nadeau et al., 2010). This *functional recovery* of *rec*‐AQP2 mutations, first identified with P262L (de Mattia et al., 2004), now shows to be wide spread and thus prompt for the introduction of a new class of recessive mutants and challenges the proposed model for NDI (Kamsteeg et al., 1999; Robben et al., 2006).

In this study, we further our investigation on NDI‐related AQP2 mutations by characterizing three new mutations (A120D, A130V and T179N) identified by our laboratory from case‐studies of affected individuals, and expected to be recessive in accordance with their location within the protein (channel structure). The characterization of previously reported A47V (Marr, Bichet, Hoefs, et al., 2002) was added to this study so as to complete the analysis of the A47V/A130V proband.

Using *Xenopus laevis* oocytes, we have performed both functional analysis and biochemical characterization of all mutants, probing both pure and coexpression conditions to determine their basic biochemical features with special emphasis on the possible recovery process through *wt*‐AQP2 hetero‐oligomerization, as done previously in our lab (El Tarazi et al., 2016; Guyon et al., 2009; Leduc‐Nadeau et al., 2010). Here again, data conflict with the standing NDI model and show that functionally recovered mutants are not the exception but rather constitute the standard outcome for such mutations, which prompted us in proposing a revised model for NDI.

## 2 MATERIALS AND METHODS

### 2.1 Ethical approval

The investigators adhere to the journals’ ethical principles and standards regarding animal handling and experimentation, and have obtained the required informed consents from all patients related to this study. All procedures regarding manipulations and treatments of animals were performed in accordance with the Canadian guidelines and ethics committee of the Université de Montréal (CDEA #20‐013). To collect oocytes, gravid *Xenopus laevis* (Nasco) were anesthetized using 2‐aminobenzoic acid ethyl ester (1.3 g/L), and ovary nodes were surgically removed and then dissected by hand to collect mature oocytes. When required, euthanasia of animals was performed by prolonged anesthesia, in accordance with institutional protocol.

### 2.2 Vectors and cRNA

All mutations for expression in oocytes were generated using site‐directed mutagenesis on the pT7Ts‐AQP2 vector, as previously described (Guyon et al., 2009; Leduc‐Nadeau et al., 2010) and constructs were validated through sequencing. For production of pT7Ts‐GFP‐AQP2, refer to (El Tarazi et al., 2016). Vectors were linearized with *SalI* from NEB, and cRNAs was synthesized using the mMessage mMachine T7 kit (Thermo Fisher Scientific).

### 2.3 Oocyte preparation, injection and maintenance

Detailed preparation of oocytes was described previously (Leduc‐Nadeau et al., 2010). Briefly, mature oocytes from gravid *Xenopus laevis* frogs were dissected by hand and treated with collagenase (17.5 mg/ml, Type 1A, Sigma‐Aldrich) in a Ca^++^ free Barth's solution (in mM: 90 NaCl, 3 KCl, 0.82 MgSO_4_, and 5 HEPES pH 7.6) to remove follicles. The oocytes were thereafter kept at 18°C in normal Barth's solution (same as above with 0.4 mM CaCl_2_ and 0.33 Ca(NO_3_)_2_) supplemented with horse serum (5%), sodium pyruvate (2.5 mM) and antibiotics (100 U/ml penicillin, 0.1 mg/ml streptomycin and 0.1 mg/ml kanamycin). The oocytes were then injected using a microinjection apparatus (Drummond Scientific) with 23 nl of water (controls) or AQP2 cRNA solutions to meet the required amount (see figure legends) and further incubated for 24 h before experimentation. As done previously (El Tarazi et al., 2016; Leduc‐Nadeau et al., 2010), care was taken to use low cRNA amounts (0.5–1 ng) so to maintain linear expression levels in both activity and protein abundances.

### 2.4 Preparation of total membrane fractions of oocytes

For descriptive methodology, see Leduc‐Nadeau et al. (2006). Briefly, total membranes were prepared by homogenizing 15 oocytes in 1 ml PBS then centrifuged at low (250× *g* for 5 min.) and high (16,000× *g* for 20 min.) speed. Final pellets were resuspended in 30 μl PBS (2 μl solution/oocyte) and processed immediately for Western blot.

### 2.5 Western blots

Western blots were performed as described previously (Bissonnette et al., 1999; 3 oocytes/sample). Samples were run on a 12% gel and transferred onto a nitrocellulose membrane. The efficiency of the overall procedure was monitored by Ponceau red staining. The membranes were first blocked with 5% BSA in TBS‐T (TBS + Tween 20, 0.1%) then incubated overnight at 4°C with α‐AQP2 (E‐2, 1:250, Santa Cruz Biotech) followed the next day by incubation for 1 h with secondary antibody (HRP‐linked goat anti‐mouse IgG #ab205719 1:10,000, Abcam). Blots were revealed, using enhanced chemiluminescence detection (SuperSignal™ West Dura Extended Duration Substrate #34076, ThermoFisher Scientific).

### 2.6 Volume measurements

Functionality of AQP2 was evaluated by water flux measurements in water‐injected (Ctrl) and AQP2‐injected oocytes incubated for 24 h. A detailed procedure is presented in previous publications (Duquette et al., 2001; Guyon et al., 2009). Briefly, the oocytes were placed in a 0.07 ml bath to allow rapid flux changes (1.2 ml per sec) and AQP2 activities were tested using a mild hypoosmotic shock (−20 mOsm by mannitol reduction). Oocyte volumes were measured in real‐time from cross‐section measurements (assuming spherical uniformity) using an inverted microscope coupled to a ×3 objective and a video camera (5 frames per sec sampling). Water fluxes (Jv) were determined from regression of the linear portions of volume traces, and specific permeability values (*P*_f_) calculated by subtracting Jv values before hypoosmotic shock to those determined after hypotonic shock. Specific AQP2 activities are presented herein as % of *wt*‐AQP2 activity.

### 2.7 Data analysis and statistics

All experiments were performed at least three times using different oocyte batches, with *n* ≥ 6 oocytes per assay. Figure legends present the specifications for data collection. Densitometry analyses was performed using Image Studio Lite software (LI‐COR).

Statistical analyses performed on individual experiments (Figures 2 and 3) represent mean ± SD values from *P*_f_ data (in % of *wt*‐AQP2). Statistical analyses combining data from *n* experiments (Figures 5 and 6) were performed using variances for each experiments and *p* values determined using one‐way ANOVA. See Statistical Summary Document S1 for individual significance (*p* values) and specifications on data collection (number experiments/oocytes per condition).

## 3 RESULTS

### 3.1 Patient

**FIGURE 1:**
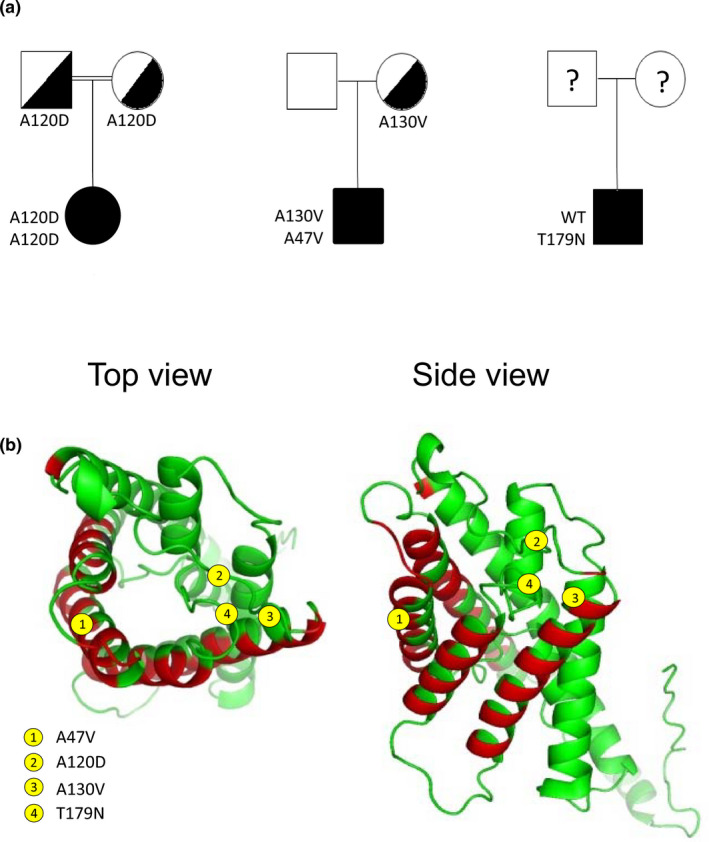
Family pedigrees and location of four mutations in study. (a) Pedigree of three families with congenital nephrogenic diabetes insipidus probands showing homozygosity for A120D, compound heterozygosity for A130V/A47V, and heterozygosity for T179N. (b) Positioning of the four mutations in study within the reported structure of AQP2 (top and side views). Sections in red represent alpha helices segments participating in interunit association.

Figure 1a presents the pedigrees for all probands with AQP2 mutations under study, and Figure 1b positions each mutation within the protein structure in accordance with the reported 3D model (El Tarazi et al., 2016). As seen in panel 1B, both A47V and A130V mutants are located in TM segments participating in interunit associations of the tetramer (red colored segments of alpha helices) while both A120D and T179N are located in adjacent extracellular loops.

The first new case‐study reports a patient of inbred ascendancy, homozygous for A120D mutation, which presented with polyuria‐polydipsia syndrome, hypernatremia and failure to thrive. Genomic DNA sequencing shows A120D heterozygosity for both parents.

The second patient was a 9‐month‐old Caucasian infant from a non‐consanguineous family presenting with reduced growth (25th percentile to 3rd percentile for height and weight at 5 months with good oral intake). Patient had reduced activity level and displayed delayed motor skills. DNA sequencing showed heterozygosity for the AQP2 gene with A47V (previously identified and characterized in Marr, Bichet, Hoefs, et al., 2002) and A130V (new mutation investigated herein). The mother carried A130V and A47V was not found in either parent (de novo in child). Confirmation of paternity was established.

The T179N mutation was identified in a 42‐year‐old obese (BMI 35) man diagnosed with hypothyroidism and uncontrolled type II diabetes, without a family record of NDI. The proband displays dilute urine (100 à 300 mOsm/kg), elevated daily water intake (15 to 20 l/day) and resistance to oral dDAVP treatment. Both glomerular filtration rate (88 ml/min) and pituitary morphology (RMN scan) were normal. Sequencing from genomic DNA confirmed wild type for V2R but indicated heterozygosity for AQP2 with T179N mutation along two inconsequential polymorphisms in exon 2 (S167S) and TGA + 20 (homozygosity). Although the patient was not detected in early age with NDI, and T179N thus most probably being a recessive variant, the possibility of a dominant trait was yet not discarded at first in account of patient's heterozygosity. We were unable to convince this patient to contact other members of his family to identify other members bearing the same mutation and expressing a polyuric phenotype.

### 3.2 Characterization of mutant AQP2 in oocytes

**FIGURE 2:**
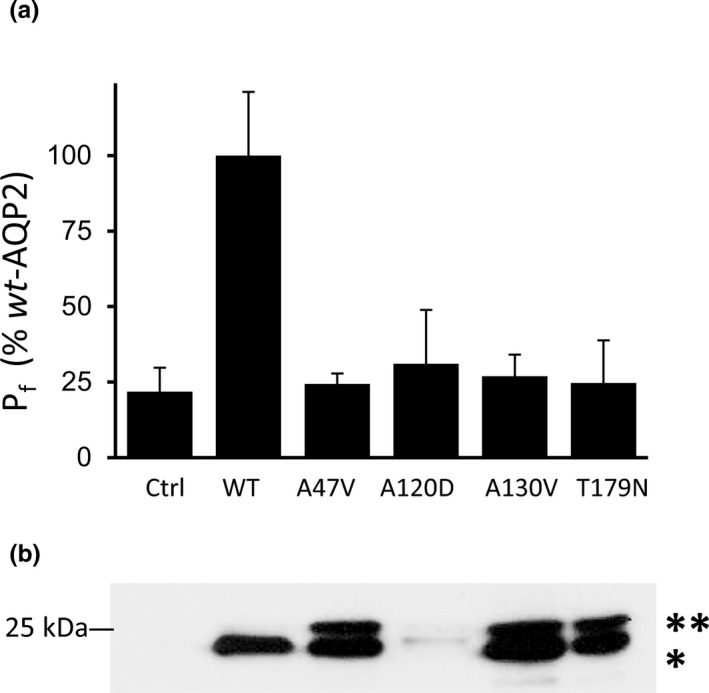
Expression of AQP2 variants in oocytes. Functionality determinations (a) along corresponding Western Blot (b) from a typical experiment (oocyte batch). Oocytes were injected with equivalent amounts of mRNAs for each AQP2 variants (0.5 ng) and incubated for 24 h prior to testing. (a) Specific P_f_ values are in % ± SD of *wt*‐AQP2 with *n* = 6 to 8 oocytes for each variant. Activity levels of all variants were similar to Ctrl (see statistics in supporting information). (b) Typical double bands (29 kDa (*) and 31 kDa (**) labeling for non‐glycosylated and high‐mannose forms, respectively, with 25 kDa MW marker indicated) are found for A47V, A130V and T179N. Note that although faint, the double bands are also found for A120D in overexposed blot.

From the start, *Xenopus* oocytes have been the favored expression system to describe NDI‐causing AQP2 mutations for both functionality and biochemical features. In this first assay, we have performed a comparative analysis of all AQP2 variants against *wt*‐AQP2 individually expressed (0.5 ng cRNA) in oocytes. As shown in Figure 2a, all mutants fail to display any significant activity, similar to control (water‐injected) oocytes.

In parallel, total membranes fractions purified from oocytes were also tested in Western blot to evaluate both protein abundances and specific profiles for each variant in study. As seen in Figure 2b, *wt*‐AQP2 displays a typical 29 kDa non‐glycosylated band, while mutant variants display profiles distinctive of *rec* forms, namely two closely‐associated bands (a high‐mannose form of 31 kDa in addition to the non‐glycosylated 29 kDa band ; Marr, Bichet, Hoefs, et al., 2002; Marr et al., 2001). Of high interest is the T179N mutation which also exhibit the double band feature typical of *rec* mutations, and not the single 29 kDa band evidenced with *dom* variants (Savelkoul et al., 2009). To further the investigation of T179N, we set out to better define its behavior against both classical *rec* (R187C) and *dom* (R254Q) AQP2 variants in co‐expression evaluations, as typically performed in our lab (El Tarazi et al., 2016).

### 3.3 Defining T179N using coexpression analysis in oocytes

**FIGURE 3:**
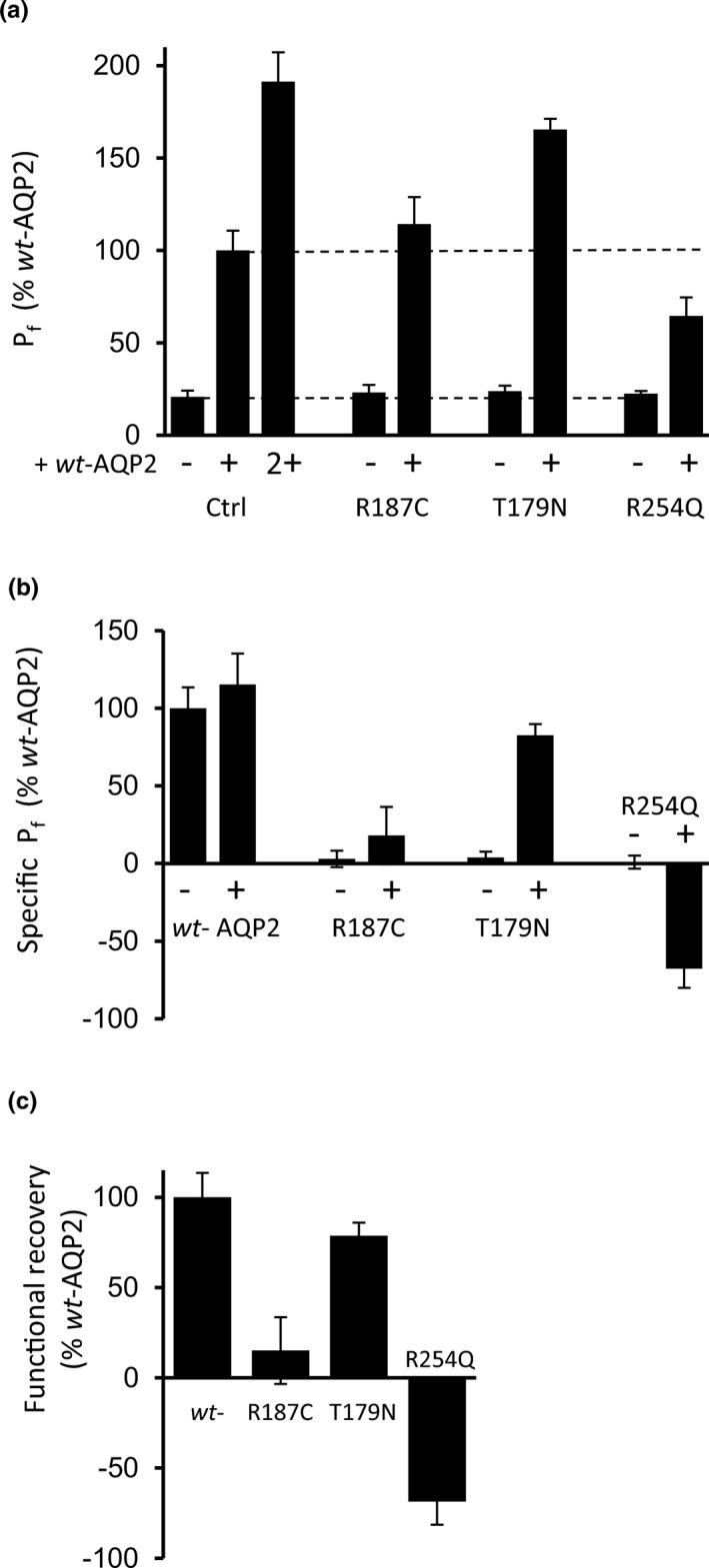
Functional analysis of T179N‐AQP2 in *wt/mutant* conditions. Oocytes were injected with 0.5 ng mRNA coding for WT, T179N, R187C and R254Q either in absence (−) or presence (+) of same amount of *wt*‐AQP2 and incubated for 24 h prior to water permeability measurements. Activities are presented in % ± SD of *wt*‐AQP2, with *n* ≥ 6 for each condition. (a) Total activity in single (−) and dual expressions (+). (b) Specific activity for single (Ctrl value subtracted from all (−) expressions), and double (*wt*‐AQP2 value subtracted from all (+) expressions) conditions. (c) Specific activity of each mutant resulting from coexpression along *wt*‐AQP2, calculated by subtracting single (−) from dual (+) values determined in (b). As expected, coexpressing with *wt*‐AQP2 does not elicit any specific activity on R187C (*p* = 0.1176, see supporting information) in opposition to T179N which display a 78 ± 7% increase in activity, similar to WT (*p* = 0.0105). On the other hand, dominant R254Q induces a 69 ± 13% reduction in activity of *wt*‐AQP2.

The property of *dom*‐AQP2 mutations to associate with *wt*‐AQP2 and impede global functionality through sequestration of *wt*/*dom* AQP2 heteromers is a key feature which can be demonstrated using oocytes, as shown by us and others (El Tarazi et al., 2016; Kamsteeg et al., 1999; Mulders et al., 1998). As mentioned earlier, we have also shown that the reverse situation also exists, this time for (at least some) *rec* variants, which may be functionally recovered through hetero‐association to *wt*‐AQP2 (de Mattia et al., 2004; El Tarazi et al., 2016; Guyon et al., 2009; Leduc‐Nadeau et al., 2010). Using this same coexpression strategy, we have tested T179N for its functionality in presence of *wt*‐AQP2, comparing data to both typical *rec* (R187C) and *dom* (R254Q) AQP2 mutants. Figure 3a presents water permeability analysis in oocytes expressing *wt*‐AQP2, R187C, T179N or R254Q either alone (− condition) or in presence of equivalent amounts of *wt*‐AQP2 (+ condition). The specific activities for each variant in both conditions were determined by subtracting either the control value from single (−) expressions, or the single *wt*‐AQP2 value from dual (+) expression conditions (Figure 3b). Finally, the discrepancies between single (−) and dual (+) expressions shown in Figure 3c represent the functional outcome attributed to each mutant when coexpressed along *wt*‐AQP2. Figure 3a shows that although the three mutants tested failed to exhibit any significant activity when expressed alone (− condition; values similar to water‐injected oocytes), their functional outcome yet varied when coexpressed with *wt*‐AQP2 (+ condition). As expected, the classical *rec* R187C variant is not impacted by the presence of *wt*‐AQP2, conversely to R254Q which hindered *wt*‐AQP2 activity by 47 ± 13%, as expected for a *dom* variant (Figure 3c). T179N, on the other hand, not only failed to inhibit *wt*‐AQP2 as expected if dominant, but rather displayed an increased functionality of 79 ± 7%, similar to several *rec* AQP2 mutations already reported (El Tarazi et al., 2016; Leduc‐Nadeau et al., 2010).

**FIGURE 4:**
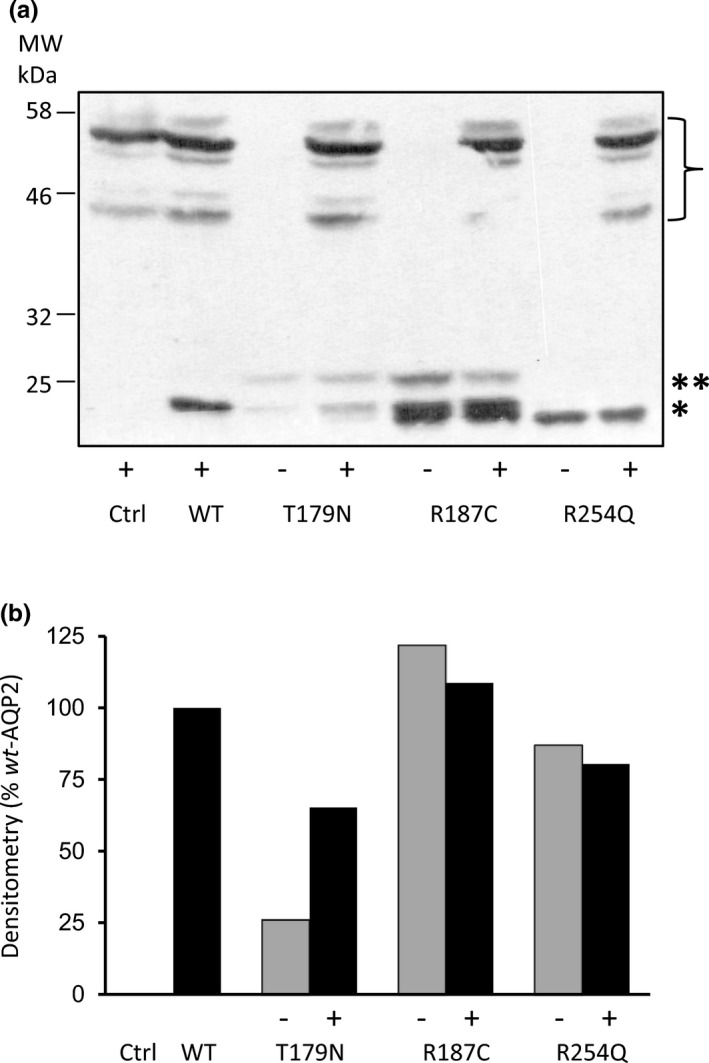
Quantization of protein abundance for AQP2 variants in *wt*/*mutant* conditions. The conditions tested in Figure 3 for functionality were also used to evaluate the protein stability for each mutant variant, this time using equimolar amount of GFP‐*wt*‐AQP2 in (+) condition in order to isolate the untagged signal of mutant forms for densitometry analysis (GFP‐*wt*‐AQP2 in bracket). Western blots in panel (a) represent the specific labeling for each mutant either in absence (−) or presence (+) of GFP‐*wt*‐AQP2. Here again, both non‐glycosylated (*) and high‐mannose bands (**) are shown. (b) Densitometry analysis for each condition displayed in (a) combining both 29 kDa (*) and 31 kDa (**) bands and presented in % values against WT.

Quite often, recovery of *rec*‐AQP2 variants is found to be associated with an increased stabilization of the mutant forms when in presence of *wt*‐AQP2. Consequently, densitometry analyses were performed in Western blots to evaluate if the abundance of T179N is altered when expressed along *wt*‐AQP2. To do so, AQP2 variants were coexpressed along the higher molecular weight GFP‐*wt*‐AQP2 (bracket, in blot) so as to discriminate signal specific to mutant forms, as done previously (El Tarazi et al., 2016). In this typical experiment presented in figure 4, coexpressing *wt*‐AQP2 only slightly reduces the density of R254Q or R187C. On the other hand, T179N density is increased by 250% (from 26% to 65% of *wt*‐AQP2, combining both 29 kDa (*) and 31 kDa (**) bands; Figure 4).

### 3.4 Testing recovery features for all AQP2 mutations in oocytes

**FIGURE 5:**
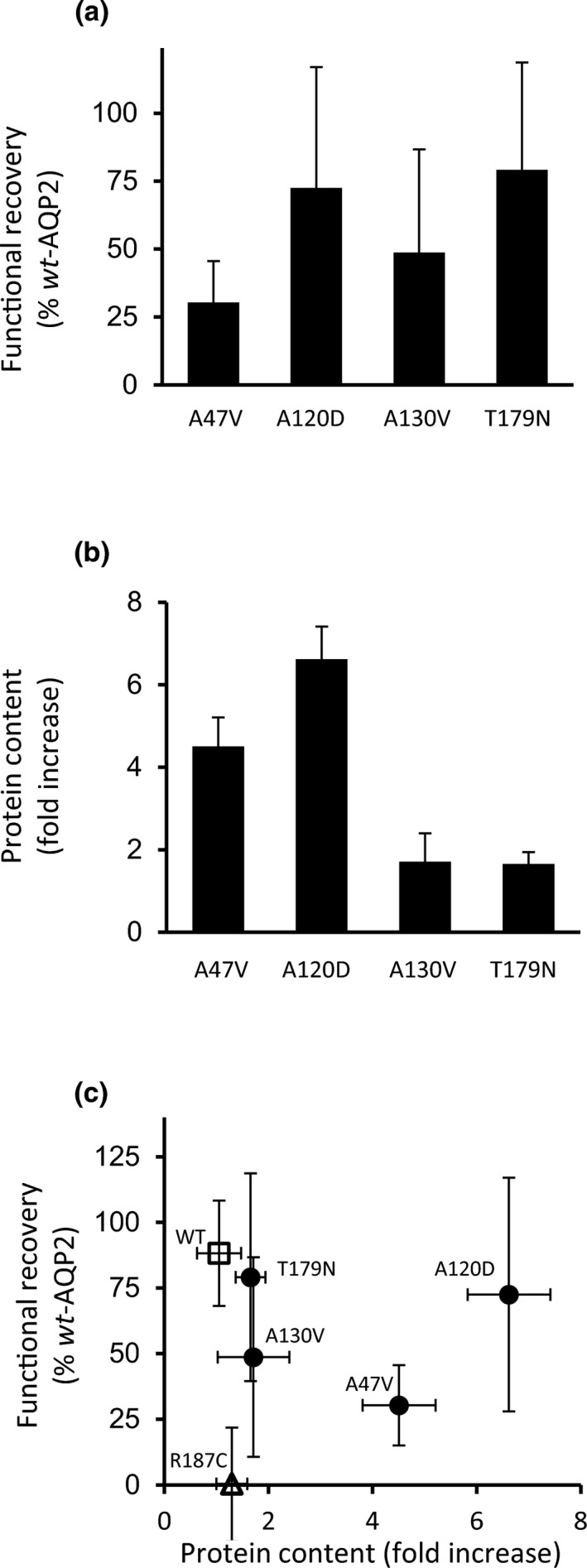
Functional recovery of four *rec*‐AQP2 mutations. Similarly to procedure in Figures 3 and 4, oocytes were injected with 0.5 ng mRNA coding for A47V, A120D, A130V and T179N either in absence (−) or presence (+) of *wt*‐AQP2 (equimolar GFP‐*wt*‐AQP2 in Western blots) and incubated for 24 h prior testing for activity (water permeability in a) and protein content (densitometry analysis of Western blot in b). (a) Recovery evaluation for each variant calculated by subtracting specific activities form single to dual (*wt* + mutant) expression conditions (similar to Figure 3c). Data represent % ± SD of *wt*‐AQP2 with *n* = 3–5 assays (see supporting information for *p* values). (b) Fold increase in protein contents (29 + 31 kDa bands) for each mutant (+ over − values, similar to Figure 4b) with *n* = 3–6 assays. (a) Plot correlating increased protein stability to functional recovery using data from panel (a) and (b), to which are added values for *wt*‐AQP2 (□) and R187C (∆).

This same strategy was next applied to all mutant variants in order to evaluate the possibility for functional recovery process in both activity (water permeability) and protein stability (densitometry analyses). As seen in Figure 5, the specific activity of each mutant is increased when in presence of *wt*‐AQP2 (Figure 5a) indicating a clear recovery process for all variants (30.2 ± 15.3%, 72.5 ± 44.5%, 48.7 ± 38% and 79.1 ± 39.6% for A47V, A120D, A130V and T179N respectively; *n* = 3–6 assays). Corresponding densitometry analyses were also performed in same expressing conditions, and increased protein stability was evaluated by calculating the ratio in band densities for each variant expressed in presence (+) of *wt*‐AQP2 over single (−) expression (Figure 5b: 4.5 ± 0.7%, 6.6 ± 0.8%, 1.7 ± 0.7% and 1.7 ± 0.3% for A47V, A120D, A130V and T179N respectively; *n* = 3–6 assays). Figure 5c correlates protein stability (densitometry fold increase) and functional recoveries (% *wt*‐AQP2) for each variant tested. As seen, both A130V and T179N variants show recovery values which correlate adequately with protein content contrary to A47V and A120D which display high fold increases in protein content. R187C (∆) presented herein depicts the unaffected *rec* mutant showing no specific increase in both functionality and protein content, similar to *wt*‐AQP2 (□) which also depicts unaltered activity and protein content.

### 3.5 Functional recovery as a general feature for *rec*‐AQP2 variants

**FIGURE 6:**
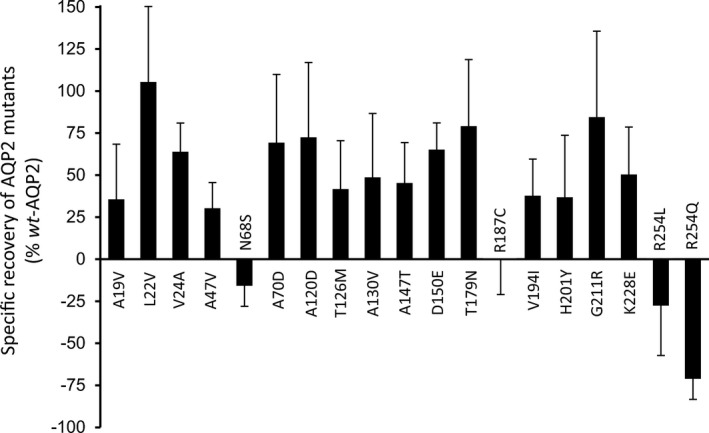
Functional recovery distribution in NDI related AQP2 mutations. The functional recovery capacities for 19 AQP2 mutations identified from NDI case‐studies was performed using the same strategy as previously, i.e. subtracting mutant activities in single expression (− condition) to that found in coexpression (+ condition, see Figure 3). As shown, mutations located in the core structure of the channel (A19V to G211R) all display clear functional recovery properties, with notable exception of N68S and R187C which are part of, or directly adjacent to, the double NPA water selectivity filter (water pore). The two last mutations studied (R254L and R254Q), located in the C‐ter regulatory segment of the channel, display *dominant negative effect* typical of *dom* mutations through inhibition of the *wt*‐AQP2 subunit(s) activity. Values are mean ± SD in % of *wt*‐AQP2, *n* = 3–5 assays (seesupporting information for *p* values).

Using the same approach, we have tested over the years several *rec*‐AQP2 variants identified in NDI patients, analyzing expressions in single (mutant alone) and dual (mutant + *wt*‐AQP2) conditions, looking at both functionality and protein expression. Figure 6 recapitulates our data for 19 *mut*–AQP2 variants, describing specific increases/inhibition properties for variants positioned throughout the entire length of the AQP2 protein. As shown, most variants depict clear increase in activity up to G211R (with notable exception of N68S and R187C), while the remaining C‐ter variants (R254L and R254Q) exhibit clear inhibition properties over *wt*‐AQP2 .

**FIGURE 7:**
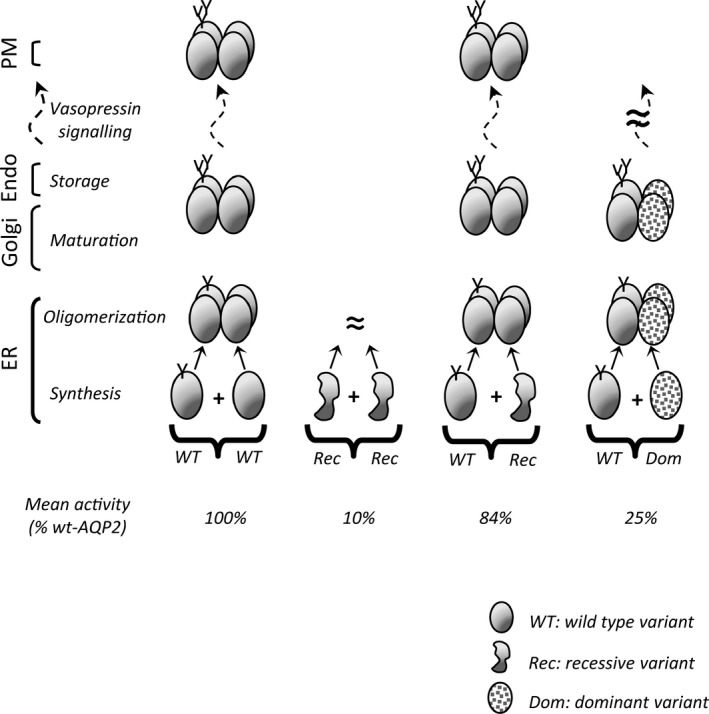
Molecular model describing AQP2‐depended NDI. In normal condition (*wt/wt*), AQP2 proteins are synthesized and assembled (tetramer) in ER before Golgi maturation and final storage in endosomes (Endo). AQP2 particles can therefore be recruited to the plasma membrane (PM) through vasopressin signaling (phosphorylation of C‐ter segment) and recycled on demand. Defective *rec* forms found within the core structure of the protein fail to produce functional tetramers in sufficient amount to support adequate level of activity, hence inducing NDI phenotype in homozygous *rec/rec* conditions (mean activity = 11% of *wt/wt* condition). Yet, aside a few exceptions (N68S, R187C), the same variants in heterozygous condition (*wt/rec*) can associate with *wt* counterparts to create functional *wt/rec* heteromers, increasing overall activity (87% of *wt/wt* condition) so to prevent NDI phenotype. Although dominant (*dom*) mutations also multimerize with *wt*, the *wt/dom* heteromers are mostly found to be sequestered (Golgi, endosomes), lowering global activity and causing NDI even in heterozygotes (25% of *wt/wt* condition).

## 4 DISCUSSION

In the pathophysiology of NDI‐causing AQP2 mutations, *rec* forms are often described as ill‐processed and non‐functional proteins, unable to associate with *wt*‐AQP2 counterparts within an active tetrameric channel (Kamsteeg et al., 1999; Tamarappoo & Verkman, 1998), although many *rec* variants of mild phenotype implying (at least) some functional tetramers are yet described (Canfield et al., 1997; de Mattia et al., 2004; Guyon et al., 2009; Leduc‐Nadeau et al., 2010; Marr, Bichet, Hoefs, et al., 2002; Mulders et al., 1997). Conversely, *dom* mutations are believed to be structurally competent as they retain the ability to associate with *wt*‐AQP2 to form heteromers (Kamsteeg et al., 1999; Robben et al., 2006), inducing NDI phenotypes in *wt*/*dom* heterozygotes through DNE (Kamsteeg et al., 1999; Katsura et al., 1995; Knoers & Deen, 2001; Marr et al., Bichet, Lonergan, et al., 2002c; Mulders et al., 1997, 1998; Tamarappoo & Verkman, 1998) and characterized by targeting issues. But for some years now, data from past studies (de Mattia et al., 2004; El Tarazi et al., 2016; Leduc‐Nadeau et al., 2010) challenged this model by describing several *rec* mutations which can oligomerize not only with itself, but also to *wt*‐AQP2, and be functionally recuperated in the process.

In this study, we set out to further our body of data relating to recovery processes of *rec*‐AQP2 variants by testing three new NDI causing mutations identified by our lab (A120D, A130V and T179N) along the previously reported A47V linked to case‐study #2. This investigation first took advantage of the debatable dominant/recessive nature of T179N to illustrate both classical DNE (inhibition) and null (recessive) actions over *wt*‐AQP2 in oocytes using R254Q and R187C variants as working models (Figure 3). As seen in data, T179N displays all the typical features of recessive variants, namely, (1) location in the core structure of the channel (last extracellular loop, Figure 1), where only recessive mutations are found, (2) a dual 29–31 kDa bands signature in Western blot (Figure 2b), and (3) strong functional recovery in both activity (*P*_f_) and protein stabilization in coexpression conditions (Figures 3 and 4). If dominant, T179N should have displayed the typical DNE features, most important of which being the inhibition of *wt*‐AQP2 function in coexpression assay (see R254Q, Figure 2), which was not the case. Hence, we do conclude in a *rec* variant for T179N as there is no evidence supporting a dominant trait. The late onset of NDI symptoms found with this specific case study is to be taken in perspective with the other health problems already reported by the proband (unhealthy 42‐year‐old proband; see patient history).

Since all variants under scope were found to be recessive, targeting issues inherent to dominant mutants, and tested essentially using cell‐line models, were thus found to be irrelevant here. This study specifically aims at characterizing the functionality of the variants expressed either alone (specific behavior of variant) or along *wt*‐AQP2 (functional recovery capacity) using the oocyte system. When expressed individually, the four mutations tested herein showed activity levels similar to control (see Figure 2), but all were found to be significantly enhanced both in activity (30.3% to 79.1% of *wt*‐AQP2) and in protein content (1.7 to 6.6 fold increase in densitometry analyses) when coexpressed along with *wt*‐AQP2. Once again, the recovery process was shown herein to be accompanied by an increased protein abundance, which strengthens the notion that functional recovery involves the stabilization of the mutant monomer(s) within mixed *wt/rec* heteromers (El Tarazi et al., 2016). Although increases in densitometry often clusters around unity or a twofold range, values reaching up to 11‐fold increases were also found for some variants (El Tarazi et al., 2016; Leduc‐Nadeau et al., 2010). Variations in fold increases are dependent on 1‐ the protein's stability in single expression (synthesis *vs* degradation rate), which gives a baseline to this evaluation, and 2‐ the protective capacity against degradation provided by association to *wt*‐AQP2 into *wt/rec* heteromers. In the present analysis, both moderate (A130V, T179N) to high (A47V, A120D) increases of protein contents are evidenced (Figure 2b).

Overall, AQP2 mutants can now be divided into three categories: 1‐ dominant mutations causing NDI in heterozygotes through DNE action (such as R254Q), 2‐ strong recessive mutations which are not improved by the presence of *wt*‐AQP2, and for which R187C has been the landmark so far (Kamsteeg & Deen, 2000; Marr, Bichet, Lonergan, et al., 2002), and 3‐ mild mutations that can be functionally recovered when coexpressed along *wt*‐AQP2, which explains the normal phenotypes in *wt/rec* heterozygotes, and consequently its recessive denomination. Figure 6 recapitulates end‐results (as presented in Figure 3c) from *wt/mutant* coexpression experiments performed in our lab throughout the last decade, showing the specific recovery/inhibitory properties for 19 NDI‐causing APQ2 variants, spanning all of the protein's structure. As shown, most of the mutants located within the core structure of the channel (from A19V to K228E) display clear recovery of activity (from 36% to 105% of *wt*‐AQP2) with notable exceptions for N68S and R187C which are part of, or immediately adjacent to the highly conserved twofold NPA (asparagine‐proline‐alanine) motifs that constitute the selectivity filter of the water channel. If excluding both N68S and R187C, the mean recovery value determined for these core‐related mutations averages 58 ± 22% of *wt*‐AQP2. Combining fractional activities from both *wt*‐AQP2 (100%) and *rec* variants (10% basal + 58% recovery = 68% of *wt*‐AQP2) to estimate a mean *wt/rec* activity level (168/2 = 84% of *wt/wt)* most certainly explains how heterozygotes manage to adequately sustain AQP2 function so to avoid NDI phenotype.

Lastly, and in accordance with structural prediction, the two C‐ter AQP2 variants tested displayed modest (−27 ± 31% for R254L) to strong (−71 ± 12% for R254Q) dominant negative effect, reducing the global AQP2 activity down to 25%, in accordance with the dominant NDI phenotype (Arthur et al., 2015; de Mattia et al., 2005).

In light of the data mustered from our many analyses of NDI‐causing AQP2 mutations, we now propose a revised model to AQP2‐dependent NDI, one where recessive mutations are essentially considered as altered AQP2 variants mostly capable of functional recovery through oligomeric associations with *wt*‐AQP2 (Figure 7). Here, *wt*‐AQP2 acts as a structural and functional support, similar to a brace or an orthotic device toward its defective *rec*‐AQP2 counterpart within the tetramer. In this concept, with exception of some strong *rec* variants (N68S, R187C), a mild or nonfunctional *rec*‐AQP2 variant causing NDI in *rec/rec* homozygotes (average activity: 10 ± 12% of *wt*‐AQP2) would gain function through *wt*‐AQP2 association in heterozygotes (*rec*/*wt*), raising its specific activity (58 ± 22% average increase) towards normal activity levels which preclude NDI phenotype (overall mean activity = 84% of *wt/wt)*. Conversely, a dominant mutation will produce *wt/dom* tetramers (at least partly) sequestered in Golgi, thus impeding the normal activity of *wt*‐AQP2 and revealing NDI phenotype (overall activity = 25% of *wt/wt*).

## 5 CONCLUSION

In this study, new AQP2 mutations characterized using *Xenopus* oocytes were found to exhibit biochemical and functional features typical of previously characterized *rec*‐AQP2 variants, the most important of which being the capacity to be functionally recovered when in the presence of its wild‐type counterpart, which explains convincingly the recessive nature of such mutations.

We suspect this remarkable feature described herein with AQP2, as also reported for other proteins (Cordat & Reithmeier, 2006; Eunson et al., 2000; Yenchitsomanus et al., 2005), to be widespread throughout multimeric protein systems. If correct, the functional recovery of impaired variants in heterozygotes could actually turn out to be a key feature to explain the overwhelmingly prevalence of recessive traits in nature (Wilkie, 1994).

## CONFLICT OF INTEREST

The authors declare no conflict of interest.

## AUTHOR CONTRIBUTIONS

*Steinke*, *Unwin*, *Rangaswamy*, and *Bichet* were responsible for clinical identification/evaluation and data collection. *Lussier*, *Matar*, *Leduc‐Nadeau*, *Da Cal*, and *Arthus* were responsible for experimentation, data collection and analysis. *Bissonnette* and *Bichet* were responsible for scientific design, data analysis, project management, and manuscript production. All were implicated in manuscript edition and review.

## Supporting information



Supplementary Material
