# Supplementary material for: Further evidence for functional recovery of AQP2 mutations associated with nephrogenic diabetes insipidus
Source: Physiol Rep. 2021 Jun 13;9(11):e14866. doi: 10.14814/phy2.14866 (PMC8198467; doi:10.14814/phy2.14866)
Supplement: Supplementary file 1 — Supplementary Material [file PHY2-9-e14866-s001.docx]

**Statistical summary document**

**Figure 2 : Single expression of Aqp2 variants in oocytes**

| **AQP2 variant:** | **Mean** | **SD** | ***n***  **(oocytes) ***** | ***p* value**  **vs CTRL** | ***p* value**  **vs WT** |
| --- | --- | --- | --- | --- | --- |
| **CTRL** | 21,82 | 7,94 | 8 | - | - |
| **WT** | 100 | 21,09 | 7 | 0.0000 | - |
| **A47V** | 24,38 | 3,46 | 8 | 0.9989 | 0.0000 |
| **A120D** | 31,02 | 17,87 | 8 | 0.7470 | 0.0000 |
| **A130V** | 26,92 | 7,16 | 7 | 0.9730 | 0.0000 |
| **T179N** | 24,70 | 14,10 | 7 | 0.9980 | 0.0000 |

**Figure 3C : Comparative evaluation of T179N-AQP2 in *wt*/*mut* conditions**

| **AQP2 variant:** | **Mean** | **SD** | ***n***  **(oocytes) ***** | ***p* value**  **vs 0** | ***p vs* WT** |
| --- | --- | --- | --- | --- | --- |
| **WT** | 100 | 13,51 | 7 | 0.0000 | 0.0000 |
| **R187C** | 15,15 | 18,52 | 8 | 0.1176 | 0.0000 |
| **T179N** | 78,72 | 7,35 | 7 | 0.0000 | 0.0105 |
| **R254Q** | -68,72 | 12,74 | 8 | 0.0000 | 0.0000 |

**Figure 5A : Functional recovery of four *rec*-AQP2 mutations – functional recovery**

| **AQP2 variant:** | **Mean** | **SD** | *****n* (assays/oocytes)** | ***p* value**  **vs 0** |
| --- | --- | --- | --- | --- |
| **A47V** | 30,32 | 15,28 | 3/24 | 0.0186 |
| **A120D** | 72,51 | 44,49 | 3/24 | 0.0000 |
| **A130V** | 48,68 | 38,01 | 5/32 | 0.0000 |
| **T179N** | 79,12 | 39,56 | 3/22 | 0.0000 |

**Figure 5B : Functional recovery of four *rec*-AQP2 mutations – protein fold increase**

| **AQP2 variant:** | **Mean** | **SD** | ***n*** | ***p* value**  **vs 1** |
| --- | --- | --- | --- | --- |
| **A47V** | 4,51 | 0,70 | 4 | 0.0000 |
| **A120D** | 6,62 | 0,80 | 3 | 0.0000 |
| **A130V** | 1,71 | 0,69 | 6 | 0.1148 |
| **T179N** | 1,66 | 0,29 | 4 | 0.2728 |

**Figure 6 : Functional recovery distribution in NDI related AQP2 mutations**

| **AQP2 variant:** | **Mean** | **SD** | *****n* (assays/oocytes)** | ***p* value** |
| --- | --- | --- | --- | --- |
| **A19V** | 35,59 | 32,80 | 3/23 | 0.0175 |
| **L22V** | 105,40 | 44,93 | 5/32 | 0.0000 |
| **V24A** | 63,89 | 17,10 | 4/30 | 0.0001 |
| **A47V** | 30,32 | 15,28 | 3/24 | 0.0186 |
| **N68S** | -15,80 | 12,20 | 5/35 | 0.9418 |
| **A70D** | 69,33 | 40,56 | 3/22 | 0.0000 |
| **A120D** | 72,51 | 44,49 | 3/24 | 0.0000 |
| **T126M** | 41,71 | 28,78 | 5/35 | 0.0002 |
| **A130V** | 48,68 | 38,01 | 5/32 | 0.0000 |
| **A147T** | 45,31 | 24,09 | 4/29 | 0.0001 |
| **D150E** | 65,18 | 15,88 | 5/36 | 0.0000 |
| **T179N** | 79,12 | 39,56 | 3/22 | 0.0000 |
| **R187C** | 0,42 | 21,42 | 5/38 | 1.0000 |
| **V194I** | 37,77 | 21,80 | 3/20 | 0.0124 |
| **H201Y** | 36,85 | 36,87 | 5/37 | 0,0018 |
| **G211R** | 84,89 | 50,32 | 5/36 | 0.0000 |
| **K228E** | 50,35 | 28,24 | 5/36 | 0.0000 |
| **R254L** | -27,59 | 29,64 | 4/24 | 0.2741 |
| **R254Q** | -71,17 | 12,18 | 3/21 | 0.0000 |

** n (oocytes)* : represent number of oocytes per assay

*** n (assays/oocytes)* : represent number of assays/overall total of oocytes. Data were assembled using variances values from individual assays.
